# *In vitro* Cartilage Regeneration Regulated by a Hydrostatic Pressure Bioreactor Based on Hybrid Photocrosslinkable Hydrogels

**DOI:** 10.3389/fbioe.2022.916146

**Published:** 2022-06-27

**Authors:** Xintong Zhao, Yujie Hua, Tao Wang, Zheng Ci, Yixin Zhang, Xiaoyun Wang, Qiuning Lin, Linyong Zhu, Guangdong Zhou

**Affiliations:** ^1^ Department of Plastic and Reconstructive Surgery, Shanghai Key Laboratory of Tissue Engineering, Shanghai Ninth People’s Hospital, Shanghai Jiao Tong University School of Medicine, Shanghai, China; ^2^ Research Institute of Plastic Surgery, Weifang Medical University, Weifang, China; ^3^ National Tissue Engineering Center of China, Shanghai, China; ^4^ Department of Cosmetic Surgery, Tong Ren Hospital, Shanghai Jiao Tong University School of Medicine, Shanghai, China; ^5^ School of Biomedical Engineering, Shanghai Jiao Tong University, Shanghai, China

**Keywords:** *in vitro* cartilage regeneration, photocrosslinkable hydrogels, hydrostatic pressure, bioreactor, nutrient permeation

## Abstract

Because of the superior characteristics of photocrosslinkable hydrogels suitable for 3D cell-laden bioprinting, tissue regeneration based on photocrosslinkable hydrogels has become an important research topic. However, due to nutrient permeation obstacles caused by the dense networks and static culture conditions, there have been no successful reports on *in vitro* cartilage regeneration with certain thicknesses based on photocrosslinkable hydrogels. To solve this problem, hydrostatic pressure (HP) provided by the bioreactor was used to regulate the *in vitro* cartilage regeneration based on hybrid photocrosslinkable (HPC) hydrogel. Chondrocyte laden HPC hydrogels (CHPC) were cultured under 5 MPa HP for 8 weeks and evaluated by various staining and quantitative methods. Results demonstrated that CHPC can maintain the characteristics of HPC hydrogels and is suitable for 3D cell-laden bioprinting. However, HPC hydrogels with concentrations over 3% wt% significantly influenced cell viability and *in vitro* cartilage regeneration due to nutrient permeation obstacles. Fortunately, HP completely reversed the negative influences of HPC hydrogels at 3% wt%, significantly enhanced cell viability, proliferation, and extracellular matrix (ECM) deposition by improving nutrient transportation and up-regulating the expression of cartilage-specific genes, and successfully regenerated homogeneous cartilage with a thickness over 3 mm. The transcriptome sequencing results demonstrated that HP regulated *in vitro* cartilage regeneration primarily by inhibiting cell senescence and apoptosis, promoting ECM synthesis, suppressing ECM catabolism, and ECM structure remodeling. Evaluation of *in vivo* fate indicated that *in vitro* regenerated cartilage in the HP group further developed after implantation and formed homogeneous and mature cartilage close to the native one, suggesting significant clinical potential. The current study outlines an efficient strategy for *in vitro* cartilage regeneration based on photocrosslinkable hydrogel scaffolds and its *in vivo* application.

## Graphical Abstract

Schematic illustration of *in vitro* cartilage regeneration using CHPC under the HP bioreactor
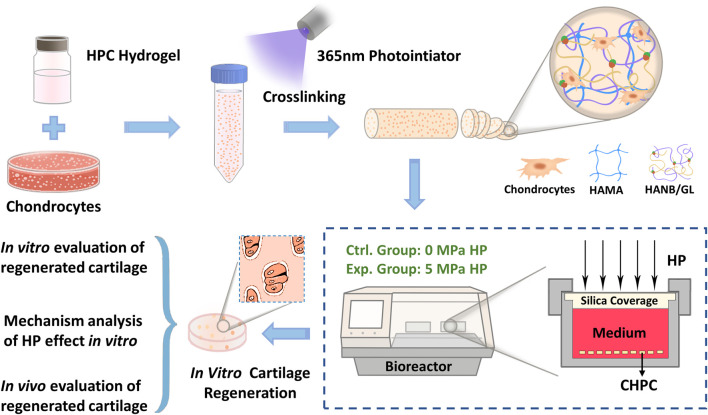

## 1 Introduction

Cartilage has a poor ability to self-repair due to its characteristic of lacking blood vessels and nerves, which makes repair of cartilage defect become great challenge (Setton et al., 1999; Jackson et al., 2001). Autologous cartilage transplantation, as one of the commonly used traditional treatment method, is limited by insufficient autologous cartilage donor and secondary trauma at the donor site (Sherman et al., 2017), while synthetic prothesis transplantation usually suffers the risks of exposure and foreign body reaction. Tissue engineering, as an emerging biotechnology, is a promising strategy to solve the problem of insufficient autologous cartilage donors. Although cartilage regeneration technology has made rapid developments in recent years, rare significant clinical breakthroughs have been achieved. The primary reason is that current cartilage regeneration techniques are mainly based on direct *in vivo* implantation of cell-material constructs, which usually fails to achieve reliable cartilage regeneration due to the influence of various disadvantageous factors, including cell loss, inflammatory responses triggered by material degradation products, trauma microenvironments caused by surgical operations, and interference of complex *in vivo* environments (Yang et al., 2012; Ou and Hosseinkhani, 2014). *In vitro* cartilage regeneration, on the contrary, can form mature cartilage prior to transplantation and thereby hopefully avoid these disadvantages, which has made it a novel and important research direction.

In the past 20 years, *in vitro* cartilage regeneration based on synthetic polymer scaffolds has significantly progressed, and *in vitro* regenerated cartilage with human-ear shape has been used clinically for ear reconstruction (Armiento et al., 2018; Zhou et al., 2018). Nevertheless, the residual polymer material in regenerated cartilage can still trigger inflammatory response in some patients and lead to heterogeneous cartilage formation, which significantly compromises clinical efficacy and greatly limits clinical translation (Shieh et al., 2004). To avoid this problem, natural biodegradable materials have become an important research topic in cartilage regeneration scaffolds due to their excellent biocompatibility, similar properties to natural extracellular matrix (ECM), and low immunogenicity *in vivo* (Qi et al., 2018). Hydrogels, as a multi-functional biomaterial which can be prepared by various natural components, have proven to be suitable scaffolds for cartilage regeneration (Cao et al., 2015; Yan et al., 2020; Zhang et al., 2020; Yuan et al., 2021). More importantly, hydrogels are the ideal vectors for cell-laden three-dimensional (3D) bioprinting, which has become an important research direction with significant prospects in the field of regenerative medicine (Markstedt et al., 2015; Wu et al., 2018). Therefore, establishing *in vitro* regeneration technology based on hydrogel scaffolds can hopefully provide an efficient research model and strategy for 3D tissue regeneration based on 3D bioprinting. However, due to difficulties in nutrient permeation and regeneration regulation, no significant breakthroughs have been achieved in the field of *in vitro* cartilage regeneration based on certain thickness hydrogels, which remains an intractable problem that requires an urgent solution.

Our previous study reported on a double-network hybrid photocrosslinkable (HPC) hydrogel with a fast gelation rate and strong mechanical strength (highly suitable for 3D bioprinting), which proved to be a suitable scaffold for *in vivo* articular cartilage regeneration (Hua et al., 2021). However, it has not been used for *in vitro* cartilage regeneration due to poor nutrient transport caused by high dense double network and static culture conditions. Previous studies have found that hydrostatic pressure (HP), as one of the most important fluid forces that chondrocytes undertake in the physiological environment of arthrosis, can effectively enhance viability, cartilage-specific gene expression, and ECM production of chondrogenic cells in traditional hydrogels (Meyer et al., 2011; Correia et al., 2012; Liu et al., 2012; Puetzer et al., 2013; Carroll et al., 2014; Jahanbakhsh et al., 2020; Aprile and Kelly, 2021). Nevertheless, the traditional hydrogels used in the above-mentioned studies are not suitable for 3D cell-laden bioprinting, and thus it is still unclear whether HP can efficiently promote *in vitro* cartilage regeneration based on HPC hydrogel scaffolds.

To clarify this issue, the following questions must be answered: 1) whether HP stimuli can improve the permeability of the HPC hydrogel scaffold? 2) does the concentration of HPC hydrogels affect the biological behavior of chondrocytes? and does HP reverse the influence of HPC hydrogel concentration? 3) can HP promote *in vitro* cartilage regeneration based on HPC hydrogels, and if so, what is the possible mechanism? 4) what is the *in vivo* fate of *in vitro* regenerated cartilage based on HPC hydrogels?

Therefore, we first tested the influence of HP on the permeability of the HPC hydrogel scaffold by immersing it in rhodamine 6G. The chondrocyte laden HPC hydrogels (CHPC) was then prepared and cultured in an HP bioreactor for 8 weeks. The biological behavior of chondrocytes and *in vitro* cartilage regeneration was evaluated to identify the effects of HP stimuli. Subsequently, the possible mechanisms of HP regulating cartilage regeneration were inferred via transcriptome sequencing. Finally, the *in vivo* fate of *in vitro* regenerated cartilage was evaluated by subcutaneous implantation in nude mice. In general, the current study uses HP stimuli provided by the bioreactor to provide a novel and efficient strategy for *in vitro* cartilage regeneration based on HPC hydrogel scaffolds.

## 2 Materials and Methods

### 2.1 Materials and Synthesis of HPC Hydrogel

Materials included hyaluronic acid (HA; Mw: 340 or 48 kDa), gelatin (GL; from porcine skin), methacrylic anhydride, sodium hydroxide, lithium phenyl-2,4,6-trimethylbenzoylphosphinate and 4-(4,6–414 dimethoxy-1,3,5-triazin-2-yl)-4-methyl morpholinium chloride (Sigma-Aldrich, United States). All the other materials were purchased from Sigma-Aldrich. In this study, the synthesis of NB-grafted hyaluronic acid (HANB) and methacrylate-modified hyaluronic acid (HAMA) were referred to our previous reports (Hua et al., 2021). Hydrogel precursors of HANB, GL, and HAMA were mixed according to the ratio of 1:1:1 to obtain the different concentration of 2%, 3%, 4%, and 6% wt% with 0.2% wt% LAP. Then, the above samples upon light (365-nm LED, 20 mW/cm^2^) irradiation were subjected to different measurements.

### 2.2 Preparation of CHPC

Chondrocytes were isolated from the auricular cartilage of 6-month-old goats (Shanghai Jiagan Biological Technology Co., Shanghai, China) and cultured as previously reported (Ci et al., 2021). During the whole experiment process, cells from three donor goats were used and cells from each donor were used in a full round of experimental process. This experiment was approved by the Ethics Committee of Shanghai Ninth People’s Hospital. The CHPC preparation process is shown in Figure 2A. Passage two goat auricular chondrocytes were mixed with liquid HPC hydrogels at a concentration of 60 million/mL and then placed in a 1 ml syringe. After irradiation with a 365 nm photoinitiator for 10–20 s, the chondrocytes were embedded in crosslinked hydrogels. The whole CHPC was then cut into 2 mm thick cylinders and cultured in our serum-free cartilage culture system (Li et al., 2019). The culture medium was replaced every 3 days.

### 2.3 Characterization of HPC Hydrogels After Cell-Loading

To measure whether encapsulated cells influenced the gelation time and mechanical strength of HPC hydrogels, HPC hydrogels with concentrations of 2%, 3%, 4%, and 6% wt% were divided into the CHPC group and the HPC hydrogel group. The inverted tube test was performed for the qualitative evaluation on the gelling property (n = 3) (Liu et al., 2016). Rheological experiments were performed on a HAAKE MARS III photorheometer with parallel-plate (P20 TiL, 20 mm diameter) geometry and 365-nm LED (20 mW/cm^2^) at 37°C (*n* = 3). Time sweep oscillatory tests were performed at a 10% strain [Controlled Deformation (CD) mode], 1-Hz frequency, and a 0.5-mm gap for 60 s (*n* = 3). Strain sweeps were performed to verify the linear response (*n* = 3). The gel point was determined as the time when the storage modulus (G′) surpassed the loss modulus (G″). The final storage modulus, calculated as the balance of rheological tests, was recorded as the final modulus of hydrogels. The compressive modulus of the hydrogels was measured by a mechanical analyzer (Instron-5542, Instron, Canton, MA) (n = 3). The hydrogel samples (wet state) were prepared as cylindrical shapes (10 mm in diameter and 3 mm in height), and the compressive speed was set to 1 mm/min. Compressive modulus was calculated from the slope of the stress-strain curve.

### 2.4 Relative Wet Weight and *in vitro* Degradation of HPC Hydrogels With Different Concentrations

The relative wet weight and *in vitro* enzymatic degradation ratio were measured by gravimetric analysis according to a previous report (Liu et al., 2016). HPC hydrogels with concentration gradients of 2%, 3%, 4%, and 6% wt% were immersed into 0.15% collagenase (Serva Electrophoresis GMBH, Heidelberg, Germany) and hyaluronidase (Aladdin Biochemical Technology Co., Shanghai) to determine the enzymatic degradation ratio *in vitro*. The freeze-dried weight of HPC hydrogels was determined after 0, 1, 4, and 7 days (*n* = 3 per group). The crosslinked HPC hydrogels were immersed in PBS (Thermo Scientific), and their wet weight was weighed after 0, 1, 4, and 7 days to calculate the relative wet weight (n = 3 per group). W_0_ represents the initial mass and W_t_ represents the mass at a certain time point, making the relative wet weight W_0_/W_t_.

### 2.5 Effect of HP on the Permeability of HPC Hydrogels

The crosslinked 3% HPC hydrogels were placed in 0.4 mg/ml water-soluble cationic dye rhodamine 6G (Damas-beta Co., Shanghai, China) dye solution. The control group was not pressurized, while the HP group was under a constant HP of 5 MPa. After immersing for 1, 3, and 5 min, the permeation of rhodamine 6G was observed under a confocal microscope at 507 nm (Vanamudan et al., 2014).

### 2.6 HPC Hydrogel Biocompatibility Testing by CCK-8 Assay

HPC hydrogel precursor (2%, 3%, 4%, and 6% wt% concentration respectively) was mixed with high glucose DMEM medium at the concentration of 5‰ (the hydrogels remained in the liquid state without crosslinking). P2 chondrocytes were then inoculated in 96-well plates at a density of 2000 cells/well, and cultured with 100 μL/well of the culture medium prepared above. The corresponding medium was replaced every other day. 10 μL CCK-8 reagent (Beyotime Biotechnology, Beijing, China) was added to each well on 1, 4, and 7 days, and incubated in the incubator for 120 min. The absorbance value at 450 nm wavelength was then measured using a microplate reader.

### 2.7 Cell Survival and Proliferation in HPC Hydrogels

CHPC was placed in phosphate buffer solution (PBS) and stained with a Calcein-AM/PI Double Staining Kit (Dojindo Molecular Technologies, Inc, Rockville, MD, United States). Live and dead cells were observed at 490 ± 10 nm under a confocal microscope. CHPC was chopped into small pieces and digested with protein kinase K overnight, and the DNA content was then detected with a Quant-iT PicoGreen dsDNA Assay Kit (Invitrogen, Carlsbad, CA, United States) to detect the cell proliferation (*n* = 3 per group). The semi-quantitative analysis of fluorescent stained images was performed with ImageJ (National Institutes of Health, United States). The relative area of live cells = live-cell area/total area of CHPC.

### 2.8 Quantitative Evaluation of *in vitro* Regenerated Cartilage

The sample volumes were measured using the drainage method, and the wet weight was obtained from an electronic balance (Mettler Toledo, Zurich, Sweden). The CHPC was chopped into small pieces and digested with protein kinase K overnight, after which the glycosaminoglycan (GAG) content was detected using the Alcian Blue method with a GAG detection kit (GENMED Scientifics Inc, Weybridge, Surrey, United Kingdom) (*n* = 3 per group). The hydroxyproline content in CHPC was determined with an alkaline hydrolysis hydroxyproline determination kit (Nanjing Jiancheng Bioengineering Institute, Nanjing, China. Since the hydroxyproline content accounts for 13.2% of the total collagen content, the total collagen content can be deduced (*n* = 3 per group).

### 2.9 Histological, Immunohistochemical, and Immunofluorescent Staining

The samples were stained with hematoxylin and eosin (H & E) and Safranin O using general histological techniques. Mouse anti-human type II collagen monoclonal antibodies (1:100 in PBS) (Santa Cruz, CA, Unied States) were used to detect the expression of type II collagen, and horseradish peroxidase-conjugated anti-mouse antibody and diaminobenzidine hydrochloride (Santa Cruz) were used for color development. Phalloidin immunofluorescent staining was performed using fluorescein isothiocyanate isomer (FITC) labeled second antibody (Solarbio), and observed at 496/516 nm under a confocal microscope. The type II collagen immunofluorescent staining was labeled by Sulfo-Cyanine3 (CY3) (Abcam) and observed at 510–560/590 nm under a confocal microscope. The concentration of antibodies used in immunofluorescent staining was 1:100, and the nucleus was labeled by 4′,6-diamidino-2-phenylindole (DAPI).

### 2.10 Real-Time Quantification Polymerase Chain Reaction (PCR)

Trizol (Invitrogen) was used to extract total RNA from CHPC at different time points, and cDNA was obtained from reverse transcription using reagents such as M-MLV and recombinant RNasin (Promega Corporation, Madison, WI, United States), as previously described (Jiang et al., 2010). The housekeeping gene encoding β-actin was used as an internal control, and the expression levels of Aggrecan (*ACAN*), collagen type II alpha 1 (*COL2A1*), and SRY-Box Transcription Factor 9 (*Sox9*) were determined. A 20 μL reaction system was prepared with a Real-Time PCR reaction solution (TaKaRa Bio Inc, Kyoto, Japan). The RT-qPCR assay was performed according to the manufacturer’s protocols (Thermo Scientific) (n = 3 per group), and the instrument was StepOnePlus Real-Time PCR System (Applied Biosystems, Foster City, CA, United States). The primers were synthesized by Invitrogen, and the sequence is shown in Supplementary Table S1. The 2^−ΔΔCT^ method was used for data analysis and calculating the relative expression levels of these three genes (Livak and Schmittgen, 2001).

### 2.11 Eukaryotic Parametric Transcriptome Sequencing

Total RNA was extracted from the samples, and a Nanodrop 2000 (Thermo Scientific) was used to detect the concentration and purity of the extracted RNA, agarose gel electrophoresis to detect RNA integrity, and Agilent 2100 (Agilent Technologies, City of Santa Clara, CA, United States) was used to determine the RIN value. Magnetic beads with Oligo (dT) were used to perform A-T base pairing with polyA to isolate mRNA from total RNA for transcriptome information analysis. Fragmentation buffers were added to randomly fragment mRNA, and separate small fragments of approximately 300 bp via magnetic bead screening. Random hexamers were applied under the action of reverse transcriptase and one-strand cDNA was reversely synthesized using mRNA as a template, followed by two-strand synthesis to form a stable double-stranded structure. End Repair Mix (Aladdin) was added to make the sticky end of double-stranded cDNA blunt. Sequencing was then performed on the Illumina platform (Illumina, San Diego, CA, United States).

### 2.12 Subcutaneous Implantation in Nude Mice

A total of eight 4–5-weeks male nude mice (Shanghai Jiagan Biological Technology Co., Shanghai, China) were used in this study, which was approved by the ethics committee of Shanghai Ninth People’s Hospital. Figure 6A shows the surgical procedure. Samples of the control group and the HP group (*n* = 8 per group) were implanted into different positions under the skin of the same nude mouse, and samples were collected after 4 weeks.

### 2.13 Suspended 3D Cell-Laden Bioprinting Based on HPC Hydrogels

The HPC gel precursor was loaded into 5-ml syringes equipped with 0.21-mm diameter needles. The syringes were then mounted into the syringe pump extruder on a Biomaker one Station (SunP Biotech, Beijing, China). Temperatures of syringes and the platform were maintained at 25°C. To fabricate one single layer of mesh-shaped construct (10.0 mm × 10.0 mm × 3 mm), each layer (0.2 mm) was printed in supporting bath (Carbomer 2020) and photo-crosslinked upon light irradiation (365 nm, 20 mW/cm^2^) within 30 s. Printing parameters were used as follows: line gap: 800 μm; layer thickness: 200 μm; photo-crosslinking time: 30 s per layer; printing speed: 10 mm/s; extrusion speed: 0.1 mm^3^/s.

### 2.14 Statistical Analysis

All quantitative data are presented as the mean ± standard deviation. After an F-test was performed on the data, a *t*-test was used to compare significances between groups. *p*-value < 0.05 was considered statistically significant (*p* < 0.05, *; *p* < 0.005, **; *p* < 0.001, ***). A total of three donor goats were involved in the study. The sample size of experiments was *n* = 3 per group, which was all noted in corresponding section. All statistical tests were performed with SPSS 21.0 (International Business Machines Corp, Armonk, NY, United States).

## 3 Results

### 3.1 Properties of Cell-Laden HPC Hydrogels Under Various Concentrations

**FIGURE 1:**
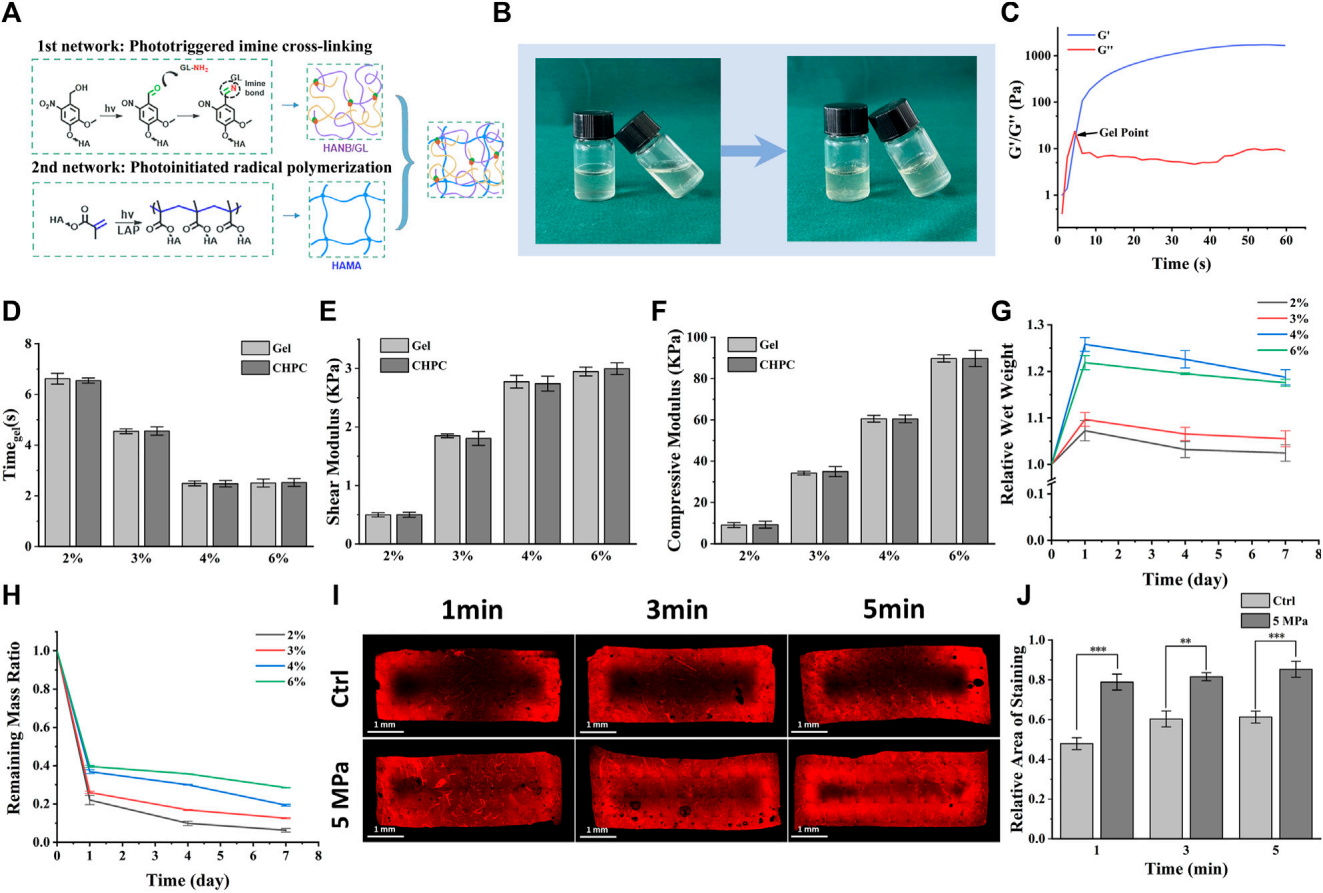
Properties of cell-laden HPC hydrogels under various concentrations. **(A)** Schematic of HPC hydrogel construction mechanism and the construction of double-networks. **(B)** Images of the HPC hydrogels before and after cross-linking under light irradiation. **(C)** Rheological measurement displaying storage modulus *(G′)*, loss modulus (G″), and gel point. **(D–F)** Gelation time **(D)**, shear modulus **(E)**, and compressive modulus **(F)** of HPC hydrogels with different concentrations loading or not loading cells. **(G)** The relative wet weight of HPC hydrogels increased as their concentrations increased. **(H)** The remaining mass in collagenase and hyaluronidase solution (0.15% w/v) indicates the enzymolysis of HPC hydrogels. **(I)** The influence of HP on rhodamine 6G permeation into HPC hydrogels. The permeable region in the HP group was obviously larger than in the control group. **(J)** Semi-quantitative graphical representation of rhodamine 6G permeation exhibiting relative areas of staining (*p* < 0.001).

Rheological analysis showed that the storage modulus quickly exceeded the loss modulus under light irradiation, suggesting the fast gelation of CHPC (Figure 1C). As shown in Figures 1D–F, gelation time and mechanical strength were related to hydrogel concentration. The gelation time decreased from 6.62 ± 0.21 to 2.48 ± 0.03 s as the concentration increased from 2% wt% to 6% wt%. Shear modulus and compressive modulus both increased as the concentration increased (shear modulus from 498.25 ± 25.73 to 3030.67 ± 49.92 kPa, and compressive modulus from 9.28 ± 0.50 to 89.74 ± 0.93 kPa). No results showed significant differences between CHPC and HPC hydrogels, indicating that the properties of HPC hydrogels were not influenced by cell loading. Figure 1G demonstrates that the water absorption increased with the hydrogel concentration and peaked on the first day due to the incomplete crosslinked network in higher concentrated hydrogel. Notably, the water absorption of 4% wt% hydrogels was higher than that of 6% wt% hydrogels, suggesting the water absorption ability was also influenced by high crosslinked density. Figure 1H shows that HPC hydrogels with lower concentrations degraded faster. Approximately 60%–80% of the initial mass was degraded on the first day, while after the first day the degradation slowed down. The effect of HP on mass transport performance of hydrogel was tested by rhodamine 6G diffusion tests and the results demonstrated that the diffusion region in the HP group was obviously larger than in the control group. Large unstained regions still existed in the control group even after 5-min immersing, suggesting that HP can obviously accelerate the mass transport of HPC hydrogels (Figures 1I,J).

### 3.2 Biological Evaluation of CHPC With Different Hydrogel Concentrations

**FIGURE 2:**
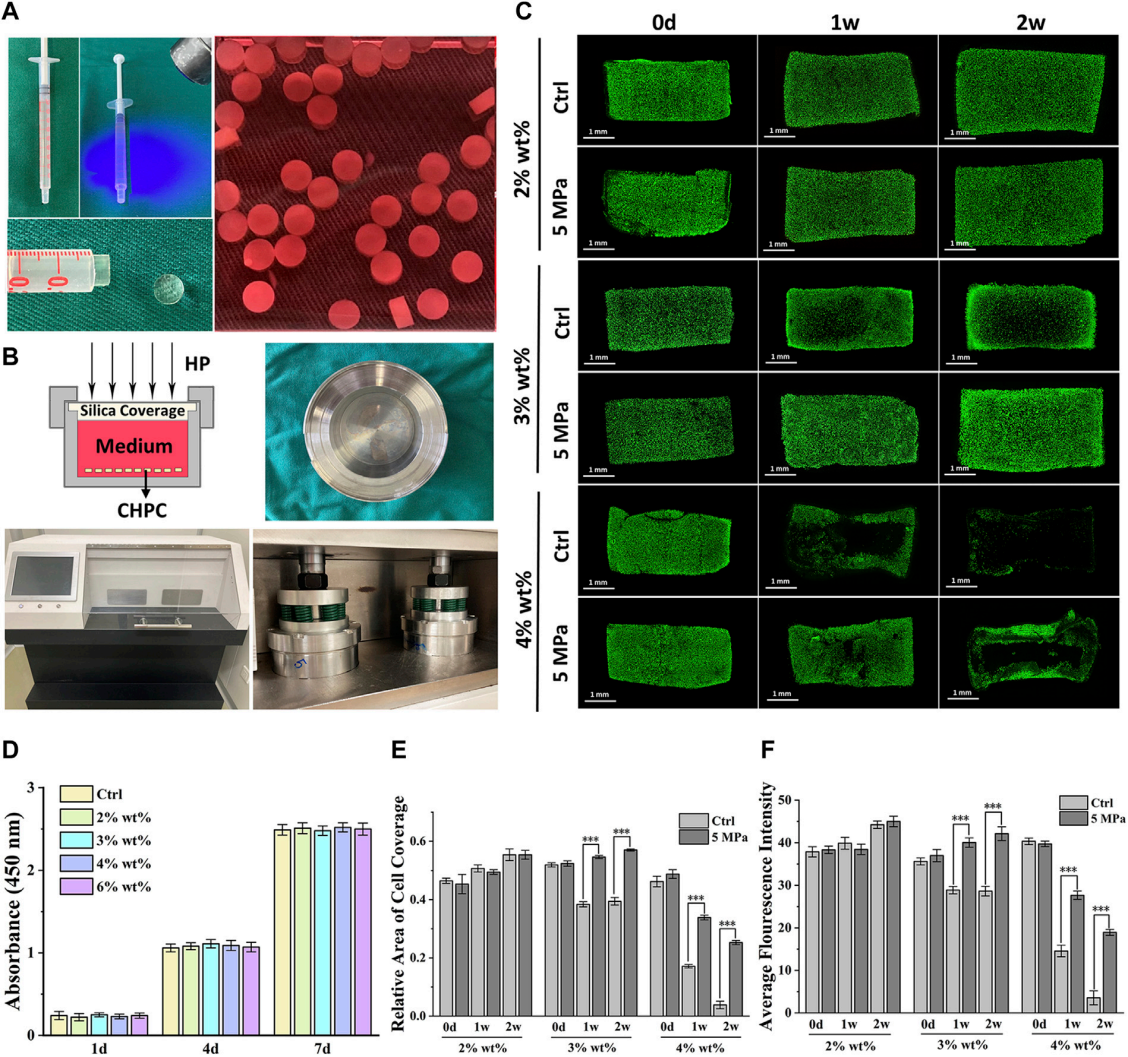
Biological evaluation of CHPC with different hydrogel concentrations. **(A)** Preparation of CHPC. The HPC hydrogels mixed with chondrocytes were placed into a syringe, crosslinked by light irradiation and cut into 2 mm cylinders, and cultured in the culture medium. **(B)** Schematic of the HP bioreactor, and images of the culture tank and HP machine. **(C)** Live and dead cell staining of CHPC after culturing for 0 days, 1 week, and 2 weeks **(D)** CCK-8 results of chondrocytes cultured in culture medium mixed with five‰ HPC hydrogels. HPC hydrogels of different concentrations in culture medium did not affect cell proliferation. **(E–F)** The relative area of live cells **(E)** and the average fluorescence intensity **(F)** (*p* < 0.001), the semi-quantitation results of Figure 2B.

The biological behaviors of chondrocytes encapsulated in HPC hydrogels were evaluated to determine the effects of various concentrations. Live and dead cell staining results of CHPC (Figure 2C) demonstrated that cells in both the control and HP groups could survive for 2 weeks at 2% wt% concentration. At 4% wt% concentration, few chondrocytes could survive for 2 weeks in the control group while some live cells could still be observed in the HP group. At 3% wt% concentration, chondrocytes in the HP group could survive and proliferate, while in the control group, the number of live cells in the central region decreased remarkably after 1 week, indicating that HP can significantly reverse the negative influence of hydrogel concentration. CCK-8 assay (Figure 2D) determined that HPC hydrogels were not cytotoxic to chondrocyte viability and proliferation. Semi-quantitation results demonstrated that the relative area of live cells (Figure 2E) and the average fluorescence intensity (Figure 2F) were significantly higher in the HP group compared to those in the control group, which was highly consistent with the results of live and dead cell staining.

### 3.3 Biological Behavior Evaluation of Chondrocytes Mediated by HP

**FIGURE 3:**
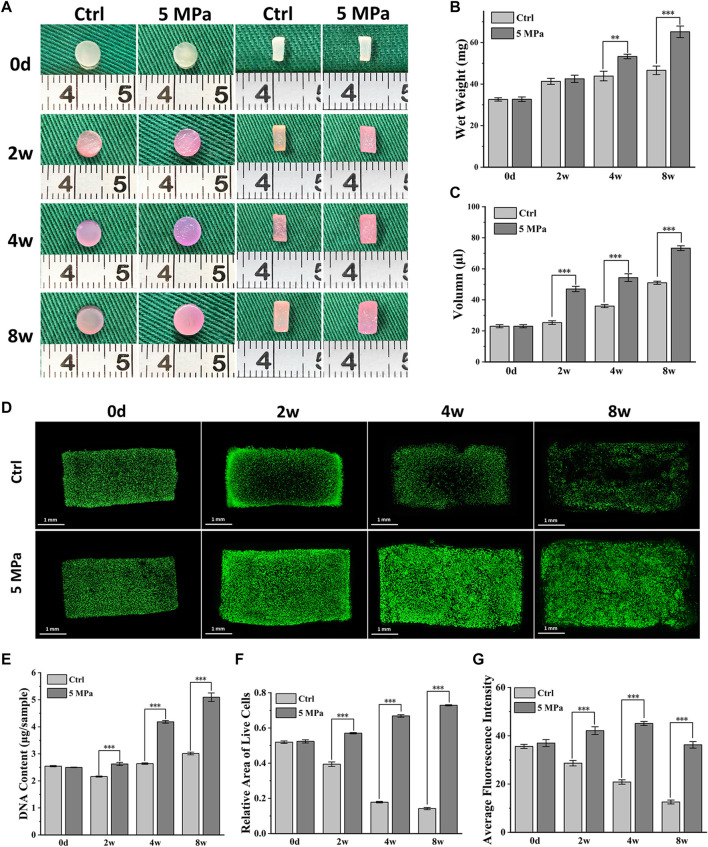
Biological behavior evaluation of chondrocytes mediated by HP. **(A)** Front and longitudinal section view of CHPC cultured *in vitro*. **(B–C)** The wet weight **(B)** and volume **(C)** (*p* < 0.05) of CHPC cultured *in vitro*. **(D)** Live and dead cell staining of CHPC cultured *in vitro*. **(E)** DNA quantification (*p* < 0.001) of CHPC cultured *in vitro*. **(F–G)** The relative area of live cells **(F)** and the average fluorescence intensity **(G)** (*p* < 0.001), the semi-quantitation results of Figure 3D.

The long-term effects of HP on the biological behaviors of chondrocytes were further evaluated. As shown in Figure 3A, the diameter and thickness of CHPC in both the control and HP groups gradually increased during 8 weeks of *in vitro* culture, though the increase was larger in the HP group. The wet weight and volume of CHPC showed similar tendencies, and significant differences between the two groups primarily emerged after 4 weeks (*p* < 0.05) and remained until 8 weeks (Figures 3B,C).

Fig. 3D displays the results of live and dead cell staining within 8 weeks. Chondrocytes in the HP group could survive and proliferate in the whole CHPC during 8 weeks of *in vitro* culture, while in the control group, the number of live cells in the central region of CHPC was low after 2 weeks. The DNA content in the HP group continuously increased and reached twice as high as initial levels after 8 weeks, while the DNA content in the control group showed little changes (Figure 3E). The semi-quantitation analysis of live and dead cell staining indicates that the relative area of live cells (Figure 3F) and average fluorescence intensity (Figure 3G) in the HP group continually increased but obviously decreased in the control group (*p* < 0.001). Surprisingly, the average fluorescence intensity in the HP group slightly decreased at 8 weeks, which was likely because the fluorescent dye had difficulty penetrating the relatively dense cartilage ECM. In addition, cell-laden HPC hydrogels could be manufactured into mesh-shaped samples (10*10*3 mm) through suspended 3D bioprinting, and live and dead cell staining results demonstrated that chondrocytes could also survive in the mesh-shaped hydrogel scaffolds after *in vitro* culture (Supplementary Figure S1).

### 3.4 Evaluation of *in vitro* Cartilage Regeneration Mediated by HP

**FIGURE 4:**
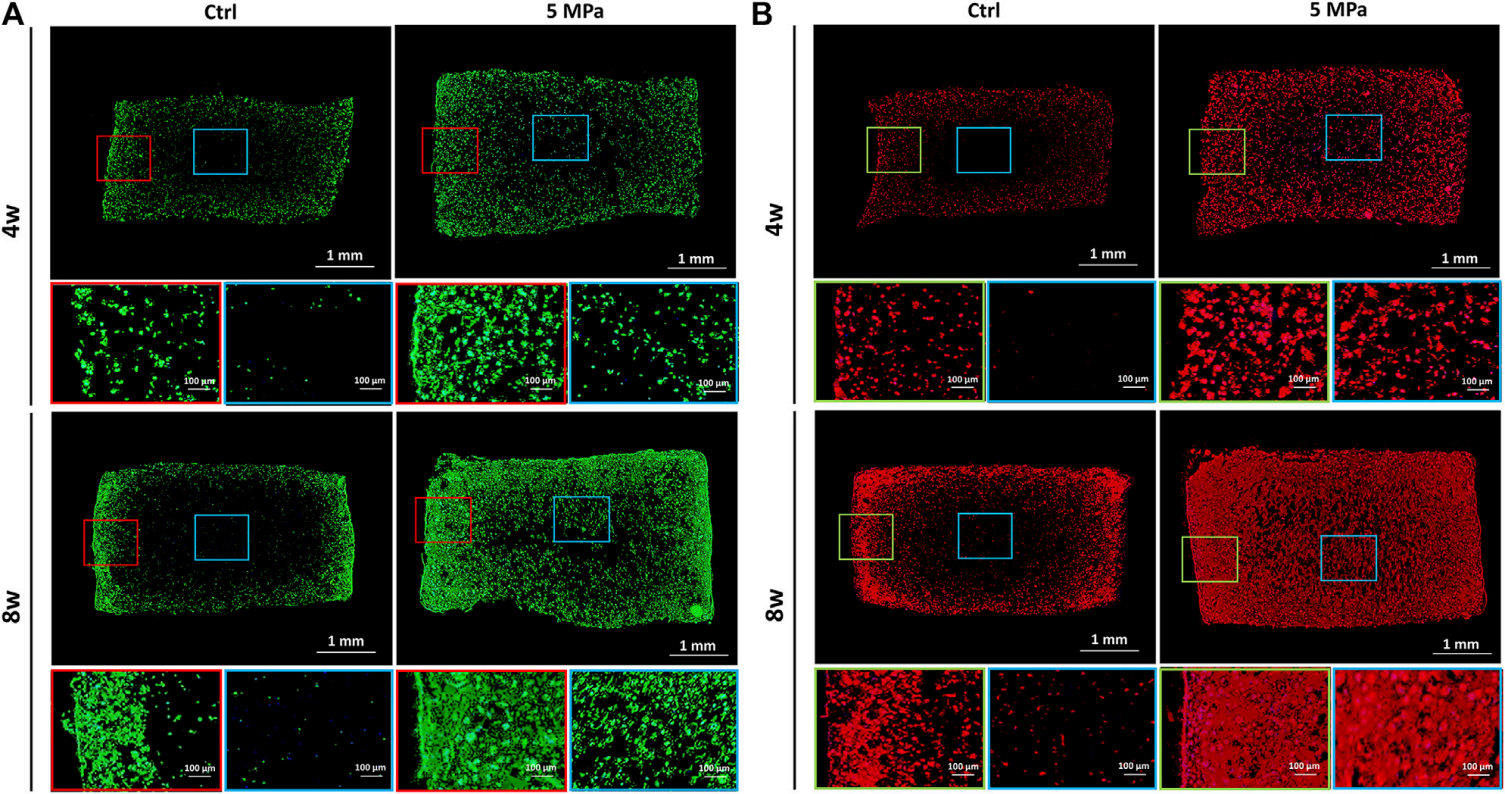
Immunofluorescent staining evaluation of *in vitro* regenerated cartilage based on HPC hydrogels. **(A)** Immunofluorescent staining of phalloidine of CHPC cultured *in vitro*. **(B)** Immunofluorescent staining of type II collagen of CHPC cultured *in vitro*. Red and green frames indicate the peripheral regions and blue frames indicate the central regions.

To visualize the cartilage ECM formation process, fluorescent staining of phalloidine and type II collagen was performed. As shown in Figure 4A, phalloidine fluorescent staining further confirmed abundant live cells throughout the CHPC in the HP group, though few live cells were observed in the central area of the control group. The cells in both groups gradually spread with *in vitro* culture time and the HP group achieved faster cell spreading, which indicates a relatively active functional status. The distributions of type II collagen at both 4 and 8 weeks in the HP group were obviously denser than in the control group, whose central regions were basically blank. The punctate deposition of type II collagen in the HP group at 4 weeks could greatly expand and merge at 8 weeks, strongly manifesting the rapid production of cartilage ECM after 4 weeks (Figure 4B), which was consistent with the tendency of wet weight and volume to increase.

**FIGURE 5:**
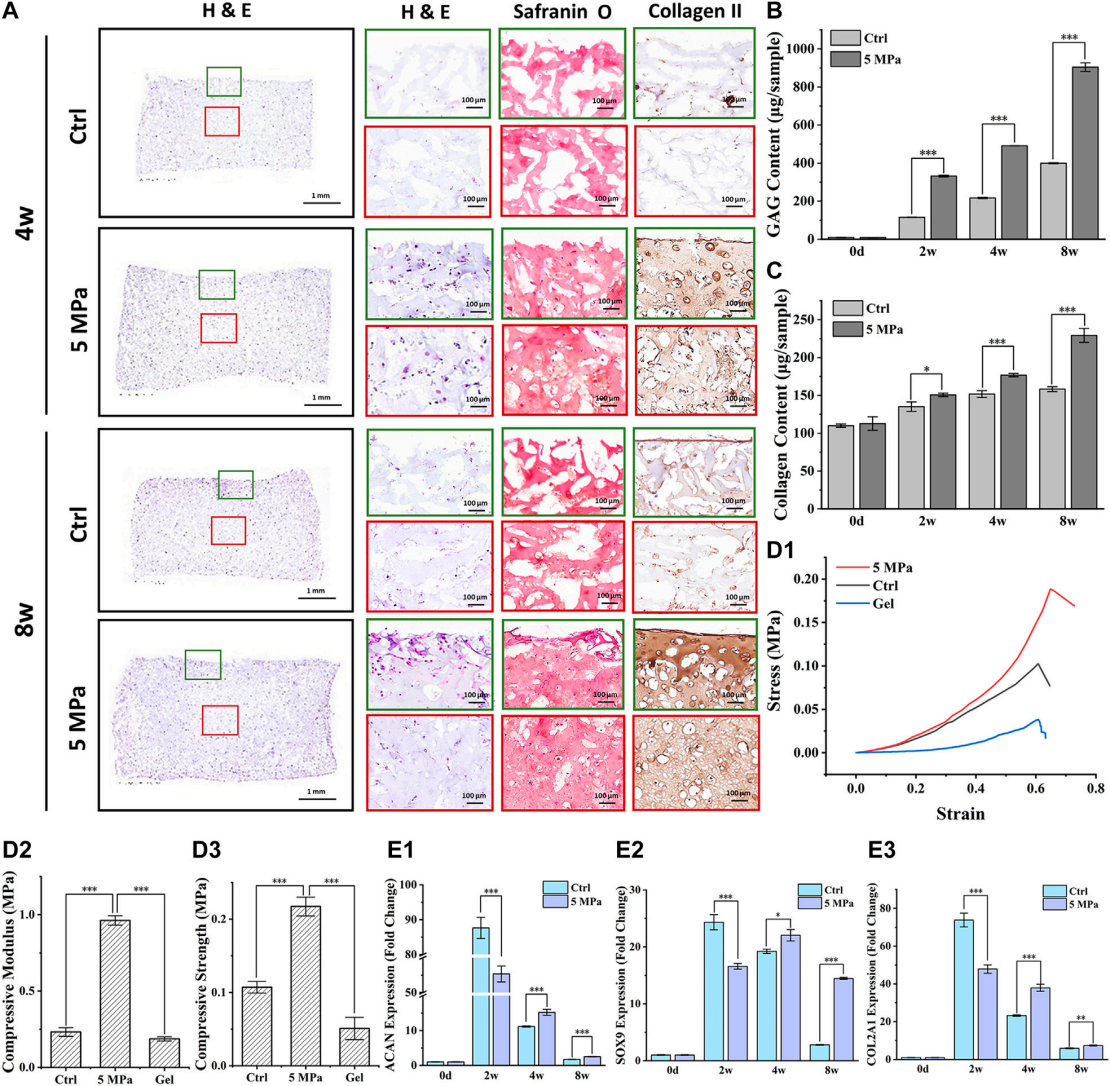
Evaluation of *in vitro* cartilage regeneration mediated by HP. **(A)** H & E, Safranin O, and type II collagen staining of CHPC cultured *in vitro*. **(B)** GAG content in the HP group and control group showed a significant difference after 1 week (p < 0.001) of *in vitro* culture. **(C)** The difference in collagen content between the two groups began to emerge after 4 weeks (p < 0.001). **(D)** Stress-strain curve **(D1)**, the compressive modulus **(D2)**, and compressive strength **(D3)** in the HP group were much higher than in the control group and HPC hydrogels (p < 0.001) during *in vitro* culture. **(E)** The fold change of *ACAN* **(E1)**, *COL2A1* **(E2)**, and *SOX9* **(E3)** expression in the HP group were higher than those in the control group after 4 weeks of *in vitro* culture (p < 0.05). Green frames indicate the peripheral regions and red frames indicate the central regions.

Consistent with the above results, histological staining further confirmed that preliminary cartilage formation with weak positive staining of type II collagen and Safranin O in the HP group occurred throughout the CHPC at 4 weeks, while relatively homogenous and mature cartilage with strong positive ECM staining and typical lacunae structures were only observed at 8 weeks in the HP group. This indicates a gradual maturation process under HP stimulus. As expected, only a small amount of weak positive staining was observed at the edge regions in the control group at 8 weeks, indicating relatively inferior cartilage formation. These results were further supported by a quantitative analysis of ECM. The GAG contents and newly synthesized collagen contents were significantly higher in the HP group at all time points than in the control group (*p* < 0.001) (Figures 5B,C). The mechanical tests demonstrated that the compressive modulus (Figure 5D2) and the compressive strength (Figure 5D3) in the HP group were much higher than in the control group (*p* < 0.001). Quantitative PCR analysis demonstrated that relative expression levels of the cartilage-specific genes *ACAN*, *COL2A1*, and *SOX9* in the HP group were significantly higher than in the control group at 4 and 8 weeks (*p* < 0.05) Figure 5E (1–3). Interestingly, we observed higher expression levels in the control group at 2 weeks, which were likely caused by relatively higher average expression levels in the control group whose cells were primarily derived from CHPC edge regions with relatively sufficient nutrients (because most cells had died in the central area), while live cells with relatively inferior nutrients in central regions of the HP group could decrease the average expression levels of the HP group.

### 3.5 Mechanism Analysis of HP Regulating *in vitro* Cartilage Regeneration

**FIGURE 6:**
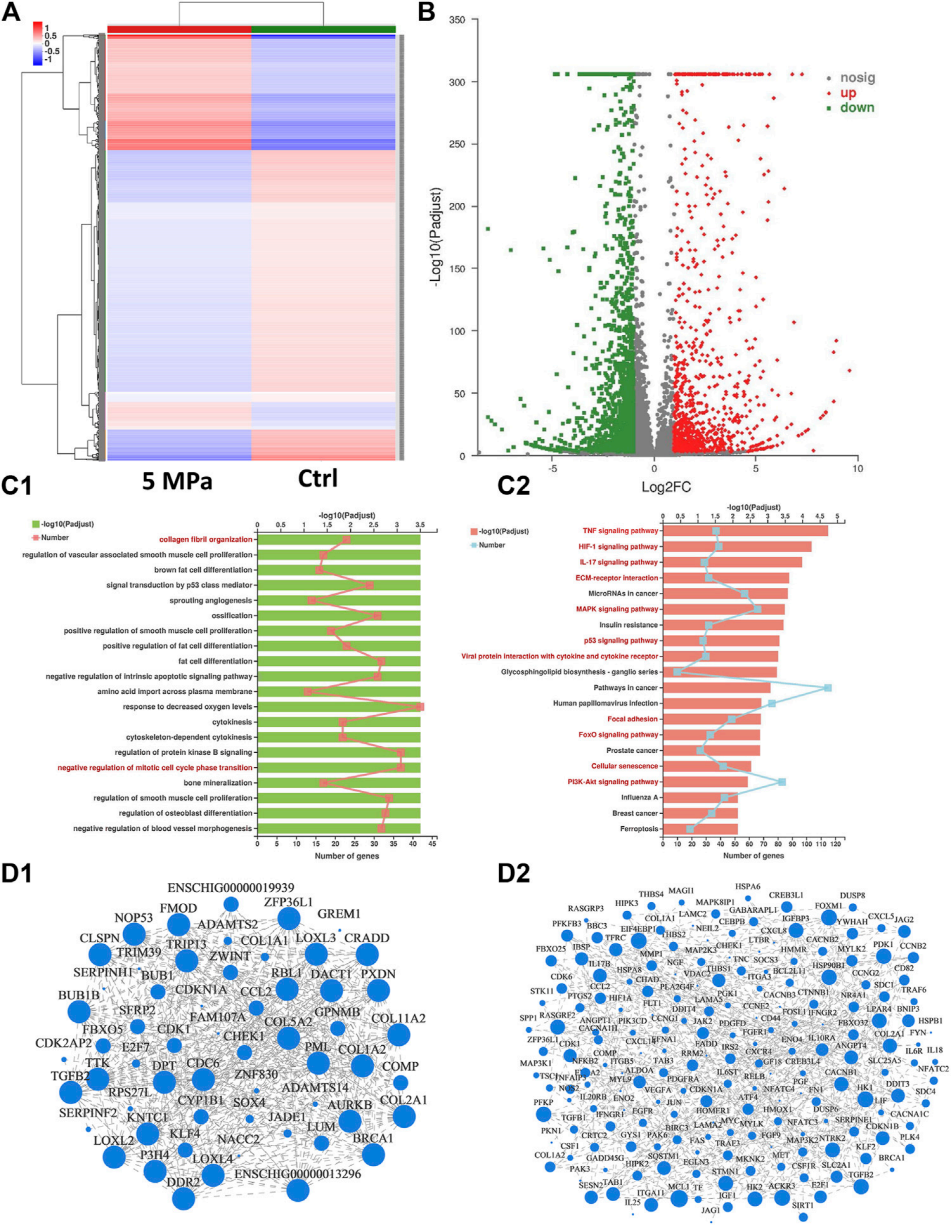
Mechanism analysis of HP regulating *in vitro* cartilage regeneration at 4 weeks **(A)** Cluster analysis heat map exhibited differences in gene expression between the HP group and the control group. The stronger the red or blue, the greater the difference in expression. **(B)** Volcano map of significance in expression levels between the HP group and the control group. A large number of genes were differentially expressed due to the effect of HP. **(C)** Functional enrichment analysis categorized differentially expressed genes into pathways referring to GO **(C1)** and KEGG **(C2)** databases. 20 potential signaling pathways with maximum probability were exhibited. Chondrocyte-related pathways were marked in red. **(D)** Visual display of the correlation between enrichment of genes referring to GO **(D1)** and KEGG **(D2)** database. The differentially expressed genes were enriched according to the potential pathways in Figure 6C.

Eukaryotic parametric transcriptome sequencing was performed to identify potential mechanisms of HP regulating *in vitro* cartilage regeneration. Figure 6A shows the cluster analysis of all differentially expressed genes between the HP and control groups. The stronger the red or blue, the greater the difference in expression. Figure 6B, the volcano map of significance in expression levels between the HP group and the control group, revealed genes that were significantly up-regulated, down-regulated, or with a non-significantly different expression. A large number of genes were differentially expressed due to the effect of HP. Additionally, compared with volcano maps of 2 weeks (Supplementary Figure S6) and 8 weeks (Supplementary Figure S2), the number of differentially expressed genes peaked at 4 weeks.

All differentially expressed genes were categorized into potential pathways referring to two genetic databases (KEGG and GO) and the potential pathways were listed from most to least possibility (Figure 6C). The top 20 potential signaling pathways were determined according to the padjust value, from which several chondrocyte-related pathways were marked in red. Figure 6D exhibited the enrichment results of differentially expressed genes included in these potential pathways, and their interrelation. For every potential pathway, all the differentially expressed genes included in it were examined referring to existing literature and the most typical genes were selected to support our conclusions. For example, the results showed that the *TP53* and *HIF1* signaling pathways which are related to cell cycle, senescence, and apoptosis, were significantly inhibited. This confirms the regulatory roles of HP on chondrocyte viability and proliferation. *COMP*, *COL2A1*, *TGFB2*, *COL11*, and their corresponding signaling pathways (such as *MAPK* and *PI3K*) were significantly up-regulated, highlighting the regulatory role of HP in ECM synthesis. *MMP*s and their corresponding signaling pathways (such as *IL*s and *TNF*) were significantly down-regulated, suggesting the negative regulation of HP on ECM catabolism. Additionally, ECM structure and mechanics-related pathways (such as collagen fibril organization, ECM organization, and focal adhesion) were also significantly activated, indicating that HP can remodel the ECM structure of the *in vitro* regenerated cartilage. Pathways related to ECM formation were only differentially expressed at 4 and 8 weeks, which was consistent with the other results. Supplementary Table S2 listed other differentially expressed genes and their corresponding pathways.

### 3.6 *In vivo* Evaluation of *in vitro* Regenerated Cartilage Based on HPC Hydrogels

**FIGURE 7:**
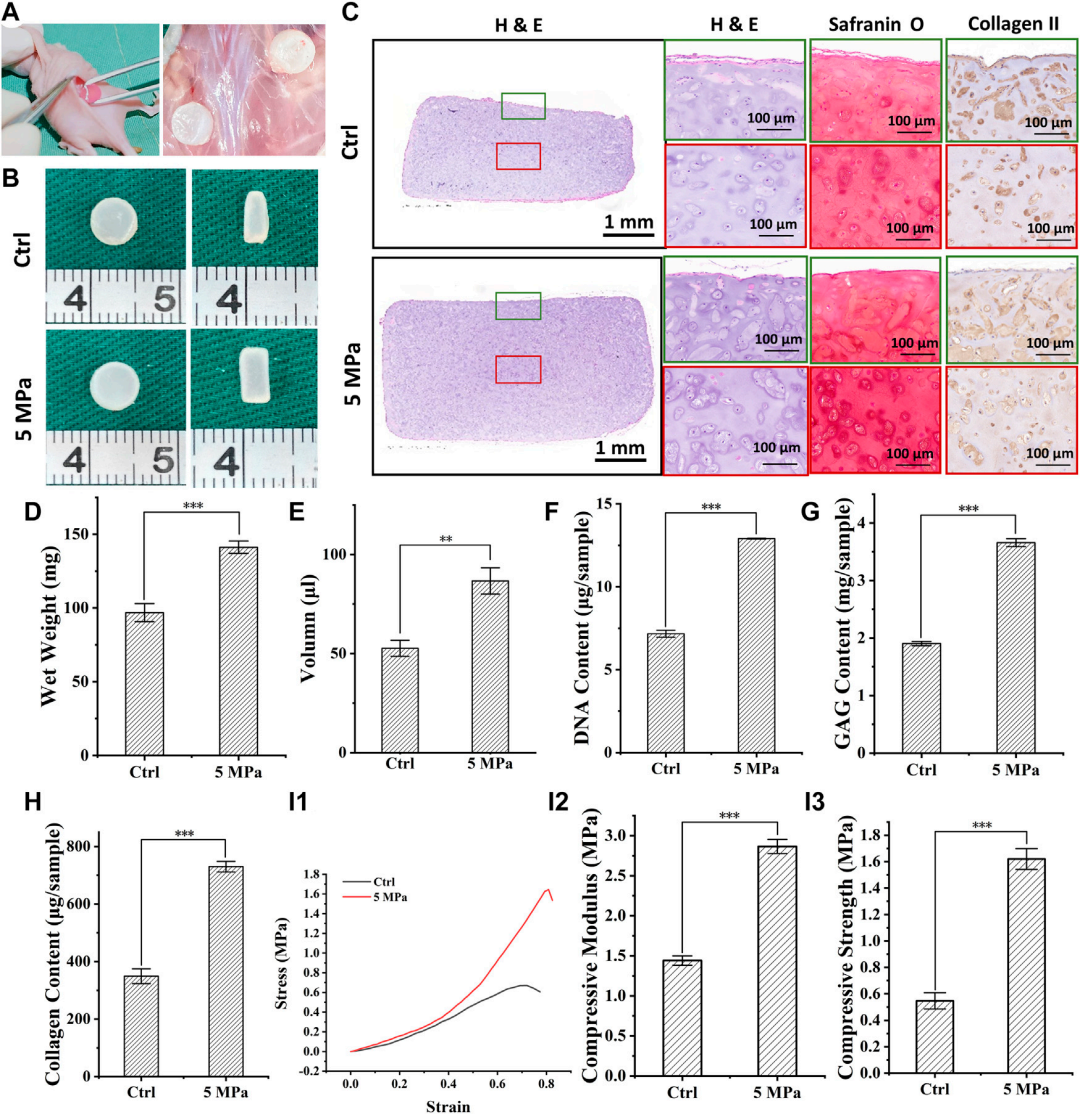
*In vivo* evaluation of *in vitro* regenerated cartilage based on HPC hydrogels. **(A)** The process of surgery and sample collection. **(B)** Front and longitudinal section view of implanted regenerated cartilage after 4 weeks **(C)** H & E, Safranin O and type II collagen staining of implanted regenerated cartilage after 4 weeks **(D–H)** Wet weight (p < 0.001) **(D)**, volume (p < 0.01) **(E)**, DNA content (p < 0.001) **(F)**, GAG content (p < 0.001) **(G)**, and total collagen content (p < 0.001) **(H)** of implanted regenerated cartilage after 4 weeks **(I)** Stress-strain curve **(I1)**, compressive modulus **(I2)**, and compressive strength **(I3)** (p < 0.001) of implanted regenerated cartilage after 4 weeks. Green frames indicate the peripheral regions and red frames indicate the central regions.

To determine the *in vivo* fate of *in vitro* regenerated cartilage, CHPC was subcutaneously implanted into nude mice after *in vitro* culturing for 8 weeks Figure 7B displays the front and longitudinal section view of the samples *in vivo* for 4 weeks. The thickness and diameter of the samples in the HP group were much larger than those in the control group. Wet weight (Figure 7D) and volume (Figure 7E) demonstrated that the regenerated cartilage in the control group only reached approximately 50–60% of those in the HP group. Histologically, the samples in the HP group formed homogeneous and mature cartilage with strong positive cartilage-specific ECM staining and typical lacunae structures. Results of the DNA content, GAG content, total collagen content (Figures 7F–H), and mechanical tests (Figure 7I) demonstrated that the regenerated cartilage in the HP group was significantly superior to the control group (*p* < 0.001). Particularly, all of these indexes significantly increased compared to before implantation, indicating that *in vivo* implantation can further promote the maturation of *in vitro* regenerated cartilage. More importantly, as shown in Supplementary Figure S3, the DNA content, ECM contents, and compressive modulus in the HP group reached 75–85% of native cartilage after *in vivo* implantation.

## 4 Discussion

Currently, *in vitro* cartilage reconstruction using hydrogels is an effective method of 3D cell-laden bioprinting (Mandrycky et al., 2016), making it an important research topic in cartilage regeneration and repair (Cipollaro et al., 2019). However, how to regenerate tissue *in vitro* with cell-laden photocrosslinkable hydrogels remains a bottleneck problem, primarily due to the difficulties in nutrient permeation caused by the dense networks and static culture conditions. This study demonstrated that HP provided by the bioreactor can significantly improve cell viability, proliferation, and *in vitro* cartilage regeneration, largely by promoting nutrient transportation and activating chondrocyte-related signaling pathways. Furthermore, after *in vivo* implantation, *in vitro* regenerated cartilage became more mature and stable, with significant differences compared to the control group that had no HP stimulus. These results provide a platform technology for various hydrogel-based tissue regeneration *in vitro*.

As the eligible vector for cell-laden 3D bioprinting, hydrogels used for *in vitro* cartilage regeneration should possess suitable mechanical, rheological, and biological properties, as well as fast gelation rate after cell loading (Schuurman et al., 2013). Our results indicate that cell loading did not influence the above properties of HPC hydrogels, and cell-laden HPC hydrogels could reach a KPa level shear modulus with a gelation time in as little as 4 s. These properties lead to the generation of tissue constructs with adequate mechanical strength and bioprinting of structures with high shape fidelity, which indicates that CHPC can serve as an operable bioink for 3D bioprinting (Gungor-Ozkerim et al., 2018).

The concentration of HPC hydrogels is one of the most important factors directly affecting the mechanical properties of the bioink and the viability of the encapsulated cells. HPC hydrogels with higher concentrations form denser internal network structures and thus have stronger mechanical strength, which is an important prerequisite for operability and 3D morphology maintenance during *in vitro* cartilage regeneration. However, there is a contradictory relationship between mechanical strength and nutrient permeability. Hydrogels with high concentrations and dense networks inevitably impede the transportation of nutrients and metabolites, which can significantly inhibit the viability and physiological activities of encapsulated cells (Shin et al., 2014; Lee et al., 2015). Our results confirmed that cell viability in HPC hydrogels significantly decreased as the concentration increased; when the concentration reached 3% wt%, the cells could hardly survive in the central area of the HPC hydrogel scaffolds after 2 weeks of *in vitro* culture. Notably, HP could significantly reverse the negative influence of high concentrations and effectively enhance cell viability in HPC hydrogel scaffolds during *in vitro* culture. Considering mechanical strength and cell viability and proliferation, 3% wt% hydrogels are appropriate for subsequent cartilage regeneration. Rhodamine 6G permeation assay further demonstrated that HP can efficiently promote the permeability of HPC hydrogels, implying that HP can improve the transportation of nutrients and metabolites. This provides a reasonable explanation for the improvement of cell viability mediated by HP.

After confirming the role of HP in cell survival, we next assessed whether HP can regulate *in vitro* cartilage regeneration based on HPC hydrogel scaffolds. Due to the special nutrient supply mechanism in cartilage, the thickness of *in vitro* regenerated cartilage is greatly limited since the formation of the outer cartilage layer can impede the nutrient and metabolite transportation of the inner region and lead to inferior inner cartilage formation (Wu et al., 2010). This phenomenon was particularly obvious during *in vitro* cartilage regeneration based on HPC hydrogel scaffolds due to the initial nutrient obstacle caused by dense networks and static culture conditions. This is an important reason why there are no studies that have assessed *in vitro* cartilage regeneration with certain thicknesses based on hydrogel scaffolds (Gardiner et al., 2007; Xu et al., 2020).

Our results demonstrated that HP can significantly promote *in vitro* cartilage regeneration based on HPC hydrogels and formed relatively homogenous cartilage at 8 weeks with a time-dependent increase of DNA content, wet weight, and volume during *in vitro* culture. On the contrary, the control group without HP stimulus only formed a small amount of cartilage in the peripheral region, and neither live cells nor cartilage formation was observed in the inner region of the HPC hydrogel scaffolds. The homogeneity, ECM deposition, and maturation of regenerated cartilage in the HP group were superior to the control group, even in the peripheral region. These results indicated that HP can significantly promote *in vitro* cartilage regeneration not only by enhancing cell viability and proliferation, but also by elevating the production and deposition of cartilage-specific ECM, which was consistent with other reports (Elder et al., 2006; Liu et al., 2013; Reinwald et al., 2015). In addition, our results indicate that the cartilage formation of CHPC presented an increasing tendency after 4 weeks.

Previous studies reported that HP could regulate some key signaling pathways (Fanning et al., 2003; Li et al., 2010; Mandal et al., 2010), and stimulate secretion of growth factors (Shin et al., 2004; Madhavan et al., 2007). To further explore the detailed mechanisms of how HP regulates *in vitro* cartilage regeneration, eukaryotic parametric transcriptome sequencing was performed and the results were analyzed. The transcriptome sequencing results indicate that the signaling pathways related to cell cycle, senescence, and apoptosis, such as *TP53* and *HIF-1*, were significantly inhibited, which strongly supports the regulatory roles of HP on chondrocyte viability and proliferation (Bates and Vousden, 1996; Zhao et al., 2018). Meanwhile, synthetic metabolism-related cartilage-specific genes (such as *COMP*, *COL2A1*, *TGFB2*, and *COL11*) and their corresponding signaling pathways (such as *MAPK* and *PI3K*) were significantly up-regulated. On the other hand, cartilage ECM catabolism-related genes (such as *MMP*s) and their corresponding signaling pathways (such as *IL17* and *TNF*) were significantly down-regulated. In addition, ECM structure and mechanics-related pathways (such as collagen fibril organization, ECM organization, and focal adhesion) were also significantly activated, indicating that HP can regulate structure remodeling of *in vitro* regenerated cartilage. These results suggest that the regulatory role of HP on *in vitro* cartilage regeneration results from synergistic action of the following factors: enhancing cell viability and proliferation, promoting ECM synthesis, suppressing ECM catabolism, and remodeling ECM structure. This reasonably explains why *in vitro* regenerated cartilage in the HP group was superior to the control group.

The *in vivo* fate of *in vitro* regenerated cartilage is another important issue that clinically concerned. Our current results demonstrated that, after *in vivo* implantation, *in vitro* regenerated cartilage in the HP group formed homogeneous and mature cartilage with significant increases in wet weight, volume, ECM contents, and mechanical properties compared to before implantation. This indicates that the *in vitro* regenerated cartilage regulated by HP can further develop and mature *in vivo.* After *in vivo* implantation, the DNA content, ECM contents, and compressive modulus in the HP group reached 75–85% of native cartilage, indicating a potential for future clinical application. The regenerated cartilage in the control group was significantly smaller than in the HP group, and the volume, total DNA content, total ECM contents, and mechanical strength only reached approximately 50–60% of the levels observed in the HP group. The most obvious differences *in vivo* between the HP and control groups can be attributed to relatively superior *in vitro* cartilage regeneration in the HP group, which possessed higher cell viability, more active ECM synthesis, and lower ECM catabolism regulated by HP. These results indicate that HP stimulus was an essential strategy for regulating *in vitro* cartilage regeneration based on HPC hydrogel scaffolds as well as its *in vivo* fate, which has significant potential in future clinical application.

## 5 Conclusion

The current research proposed a new strategy for *in vitro* cartilage regeneration based on photocrosslinkable hydrogel scaffolds that are suitable for 3D bioprinting. Our results demonstrate that HP provided by the bioreactor can significantly promote the nutrient permeability of photocrosslinkable hydrogels, and effectively enhance the viability and proliferation of cells during *in vitro* culture. HP also significantly elevated the production and deposition of cartilage-specific ECM. Further analysis of the mechanisms indicates that HP can regulate *in vitro* cartilage regeneration by inhibiting senescence and apoptosis, promoting ECM synthesis, suppressing catabolism, and ECM structure remodeling. More importantly, the evaluation of *in vivo* fate indicated that *in vitro* regenerated cartilage in the HP group can further develop and mature after implantation and form homogeneous and mature cartilage close to native cartilage. This indicates that it has significant potential for future clinical application. While *in vitro* cartilage regeneration based on 3D cell-laden bioprinting and cartilage defect repair in large animals require further investigation, this study outlines an efficient and essential method for *in vitro* cartilage regeneration and its *in vivo* applications using photocrosslinkable hydrogel scaffolds.

## Data Availability

The original contributions presented in the study are included in the article/Supplementary Material, further inquiries such as the entrance to the sequencing database can be directed to the corresponding authors.
