# Supplementary material for: In vitro Cartilage Regeneration Regulated by a Hydrostatic Pressure Bioreactor Based on Hybrid Photocrosslinkable Hydrogels
Source: Front Bioeng Biotechnol. 2022 Jun 27;10:916146. doi: 10.3389/fbioe.2022.916146 (PMC9273133; doi:10.3389/fbioe.2022.916146)
Supplement: Supplementary file 1 [file DataSheet1.docx]

Supplementary Material

# Supplementary Figures and Tables

## Supplementary Figures

**
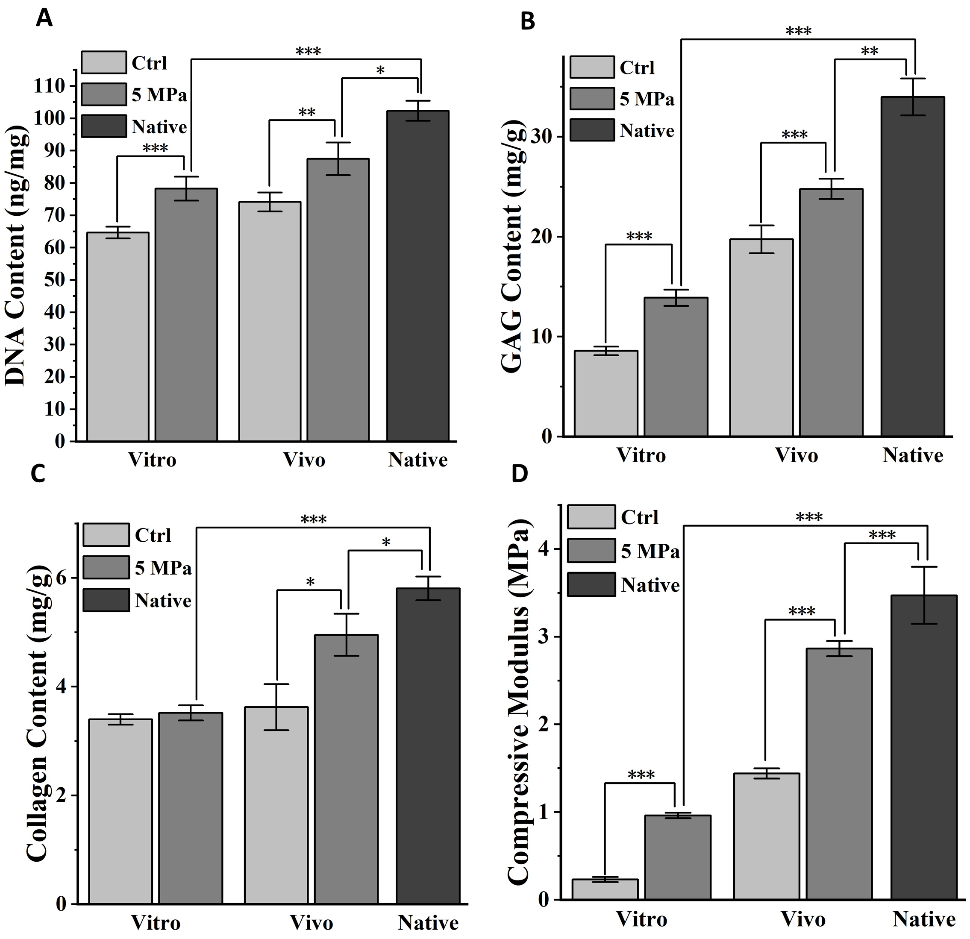
**

**Supplementary Figure 1.** Comparative evaluation between regenerated cartilage and native cartilage. **(A-D)** DNA, GAG, collagen, and compressive modulus in the control group (*in vitro* 8 weeks and *in vivo* 4 weeks), HP group (*in vitro* 8 weeks and *in vivo* 4 weeks), and native cartilage (p<0.001).


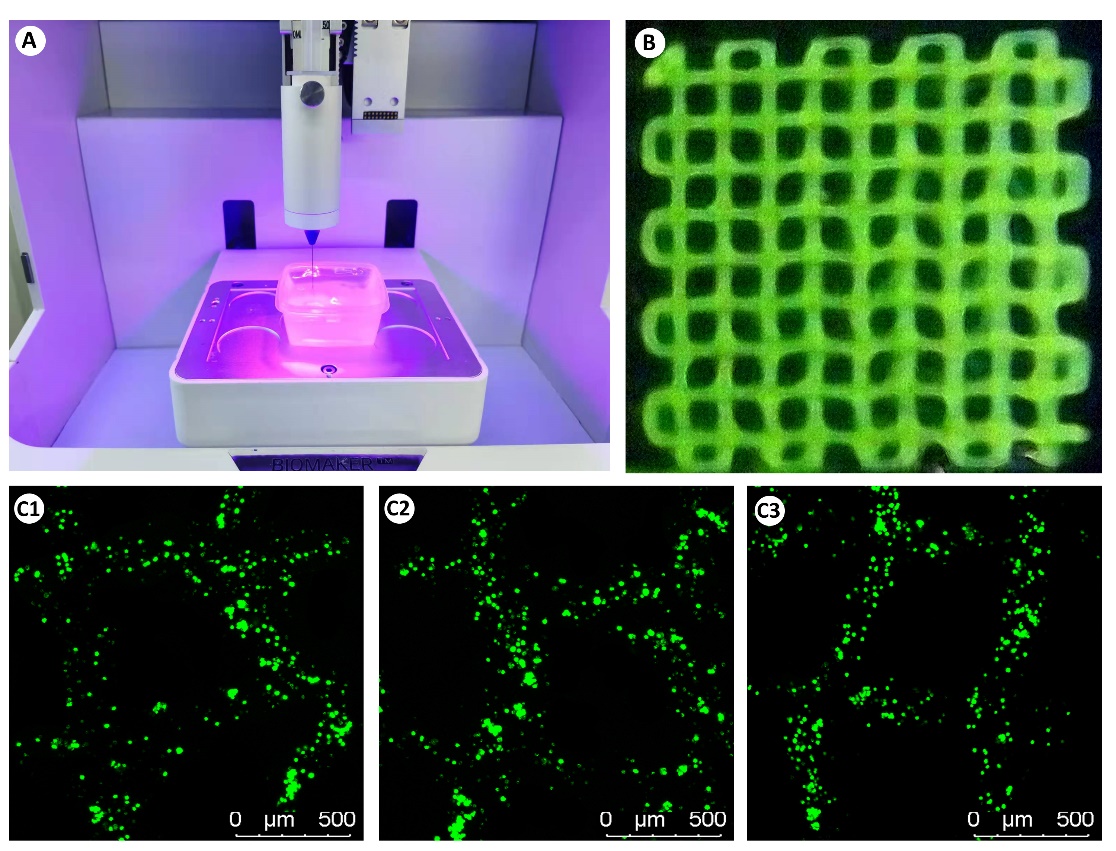


**Supplementary Figure 2.** Suspended 3D cell-laden bioprinting based on HPC hydrogels. **(A)** The process of suspended 3D bioprinting. **(B)** The mesh-shaped structure (FICT-stained) after 3D bioprinting. **(C)** Live and dead cell staining of chondrocytes in mesh-shaped HPC hydrogel scaffolds after days 1 **(C1)**, 4 **(C2)**, and 7 **(C3)** cultured *in vitro*.


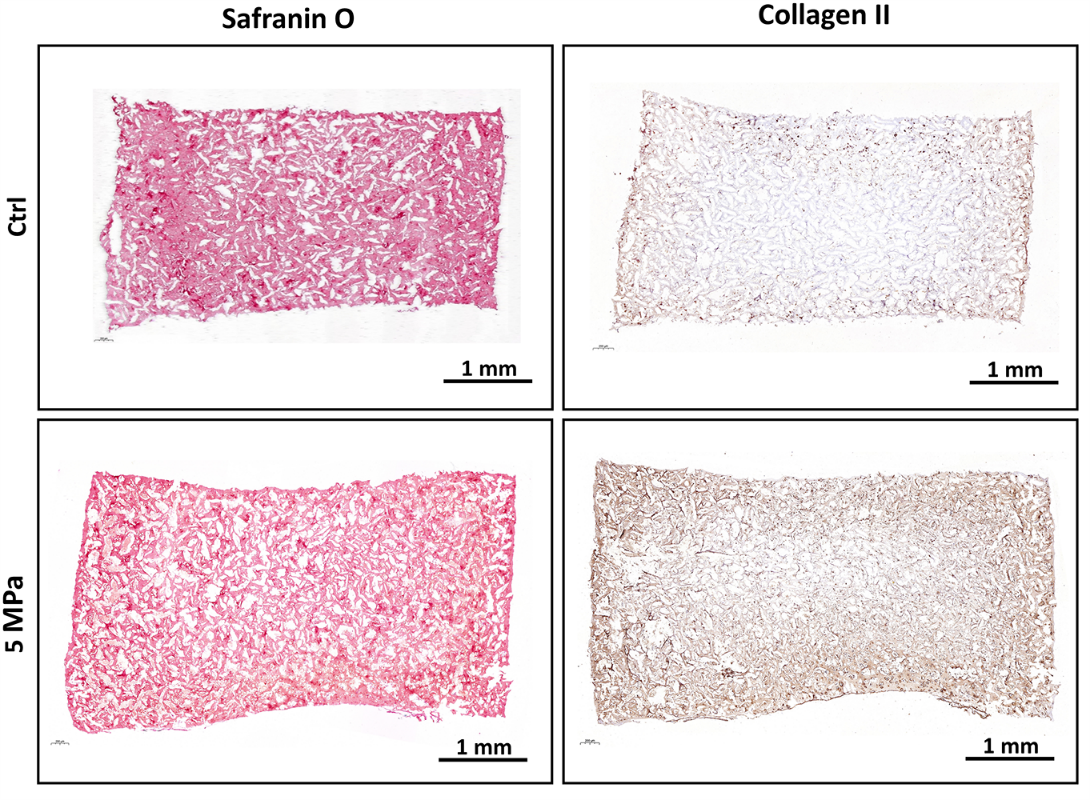


**Supplementary Figure 3.** Safranin O and type II collagen staining of implanted regenerated cartilage after 4 weeks.


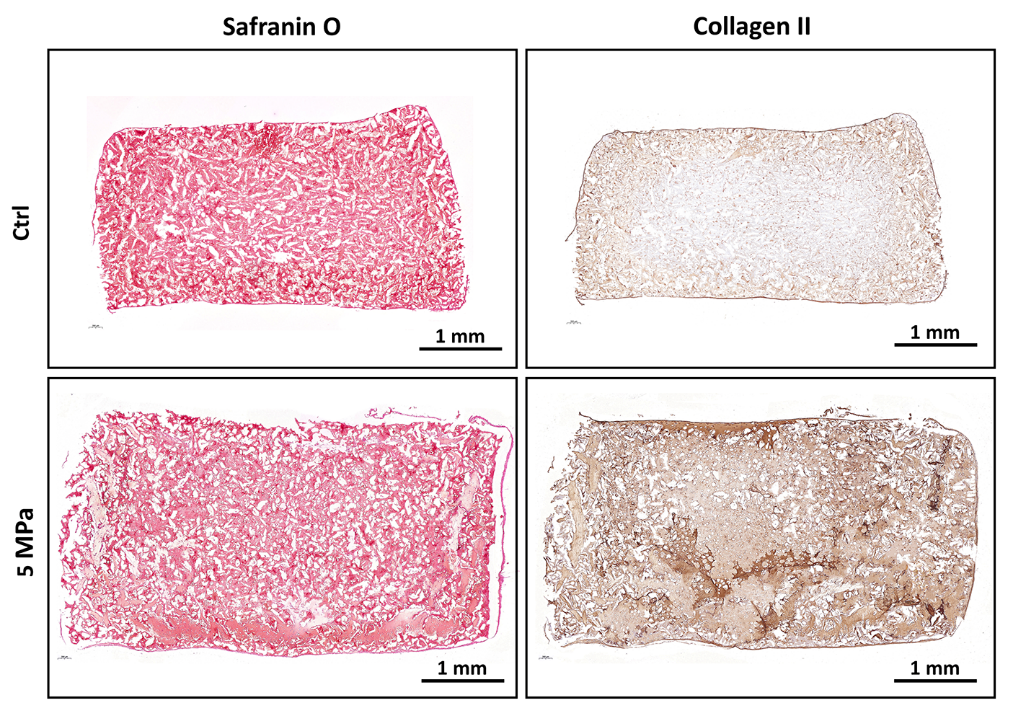


**Supplementary Figure 4.** Safranin O and type II collagen staining of implanted regenerated cartilage after 8 weeks.


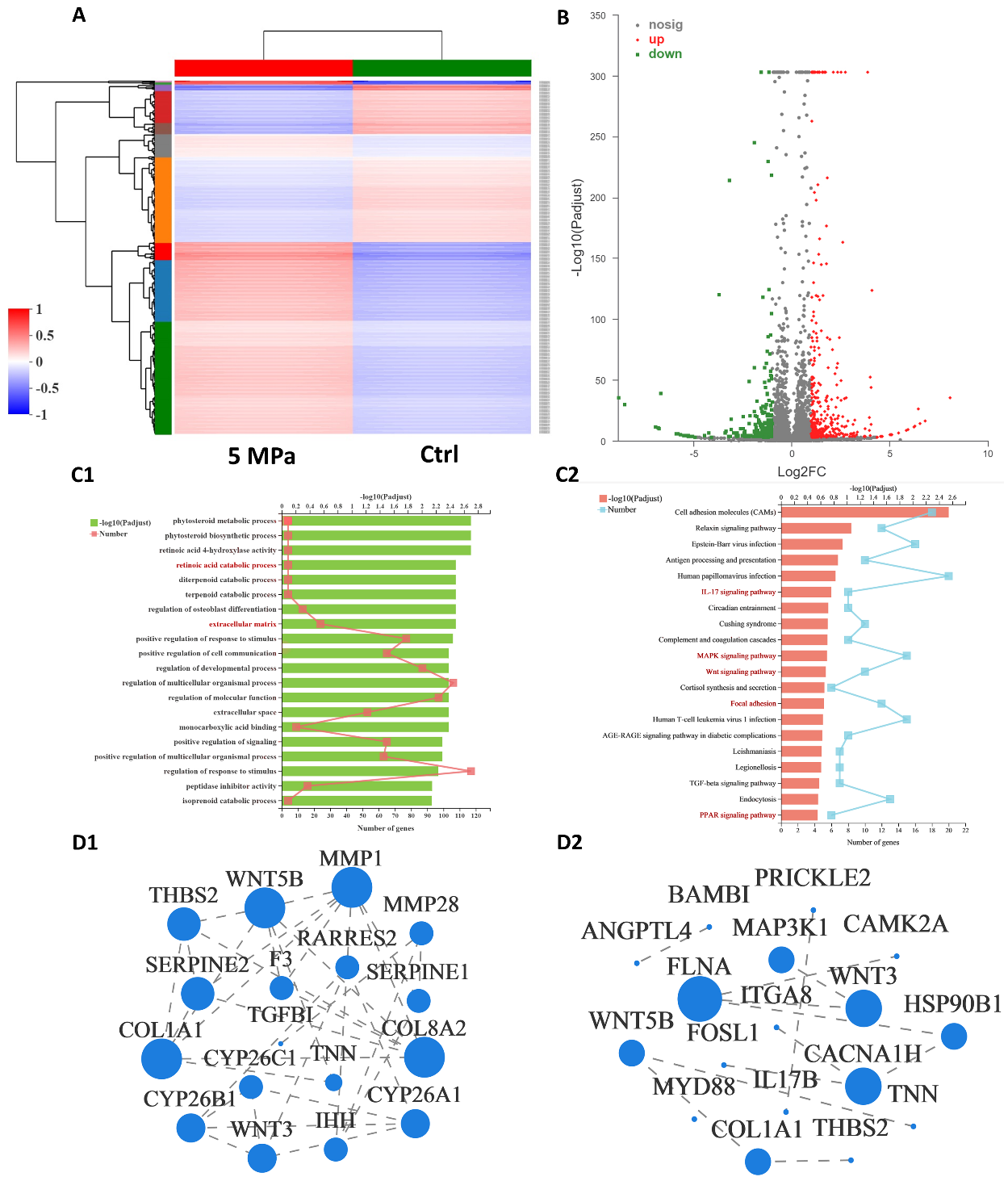


**Supplementary Figure 5.** Mechanism analysis of HP regulating *in vitro* cartilage regeneration at 2 weeks. **(A)** Cluster analysis heat map displays differences in gene expression between the HP group and the control group. **(B)** Volcano map of significance in expression levels between the HP group and the control group. **(C)** GO **(C1)** and KEGG **(C2)** functional enrichment analysis. **(D)** Visual display of the expression correlation between GO **(D1)** and KEGG **(D2)** enrichment genes.


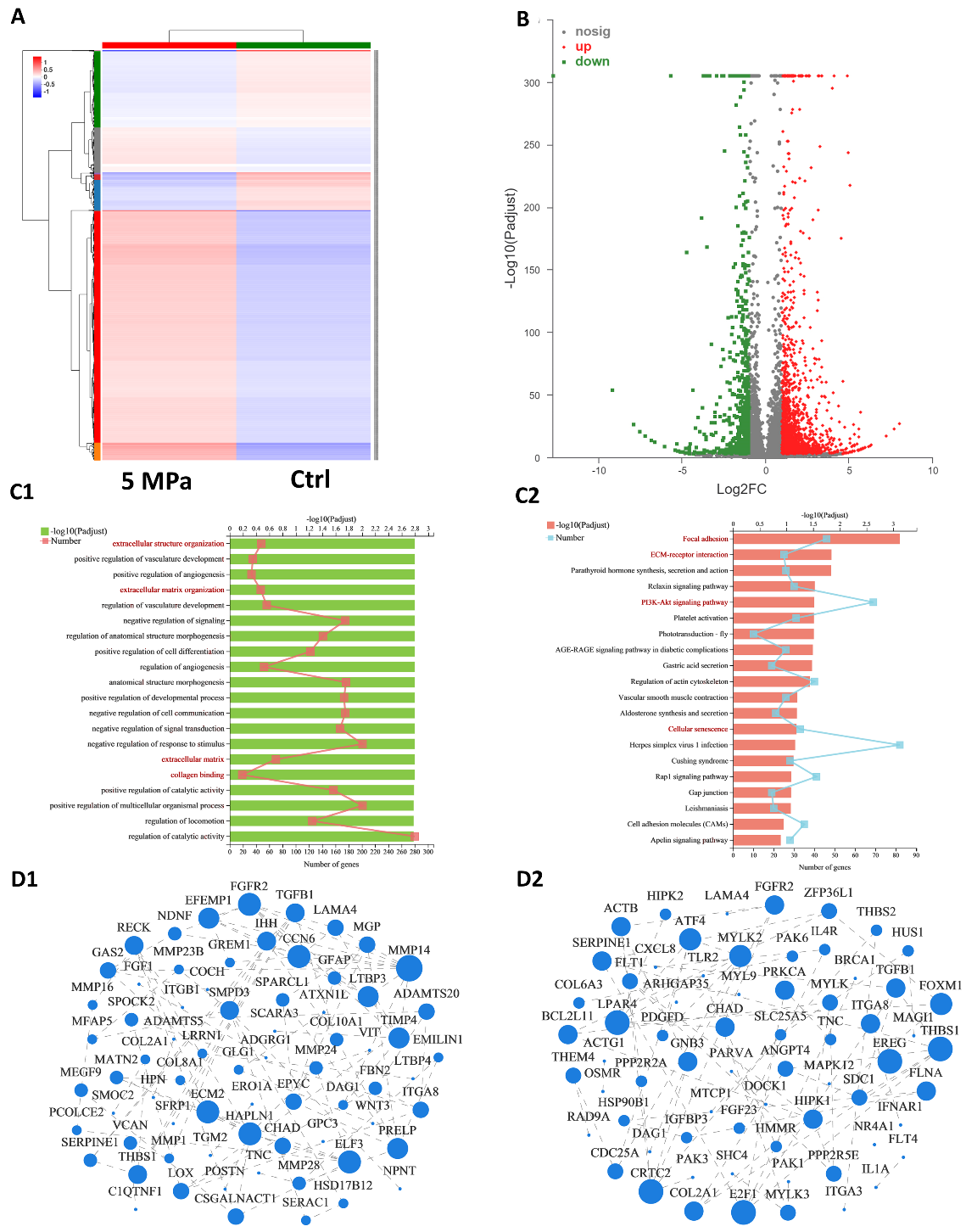


**Supplementary Figure 6.** Mechanism analysis of HP regulating *in vitro* cartilage regeneration at 8 weeks. **(A)** Cluster analysis heat map displays differences in gene expression between the HP group and the control group. **(B)** Volcano map of significance in expression level between the HP group and the control group. **(C)** GO **(C1)** and KEGG **(C2)** functional enrichment analysis. **(D)** Visual display of the expression correlation between GO **(D1)** and KEGG **(D2)** enrichment genes.


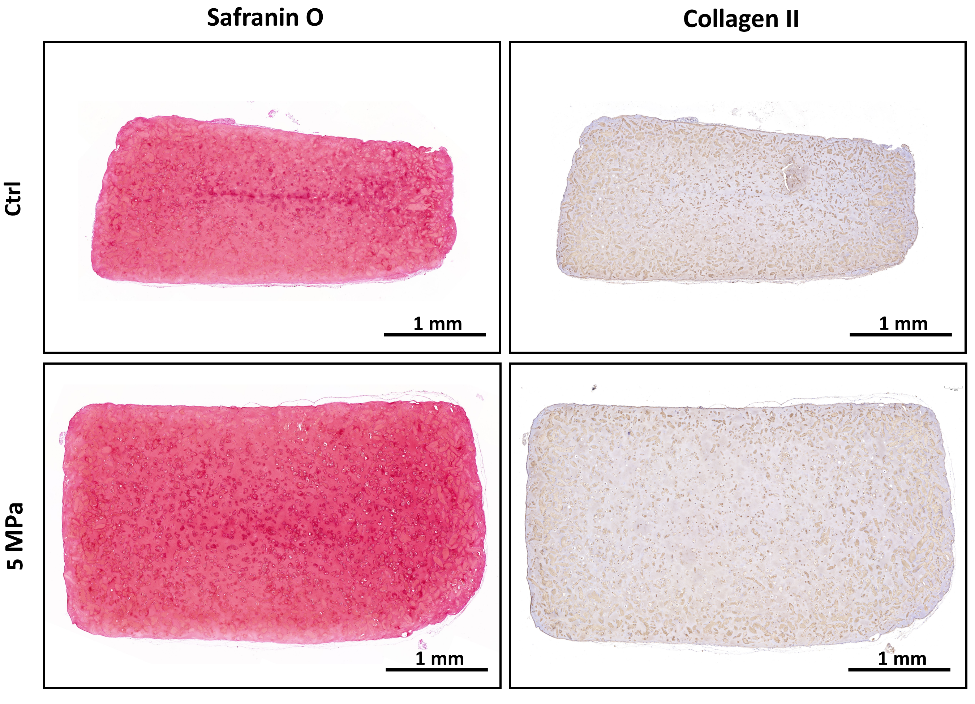


**Supplementary Figure 7.** Safranin O and type II collagen staining of implanted regenerated cartilage after 4 weeks.

## Supplementary Tables

**Supplementary Table 1.** Primer sequences of related genes.

| **Genes** | **Primer Sequences (5'-3')** |
| --- | --- |
| *COL2A1* | GCATTGCCTACCTGGACGAAGTCACAGTCTCGCCCCACTTAC |
| *SOX9* | GCATTGCCTACCTGGACGAAGTCACAGTCTCGCCCCACTTAC |
| *ACAN* | GCATTGCCTACCTGGACGAAGTCACAGTCTCGCCCCACTTAC |

**Supplementary Table 2.** Differentially expressed genes regulated by HP and related pathways.

| **Genes** | **Differential Expression Regulated by HP** | **Related Pathways** |
| --- | --- | --- |
| *COL11, COMP, TGFB2, COL2A1* | up | Collagen Fibril Organization |
| *FOXM1* | up | P53 signaling pathway to cell senescence and apoptosis |
| *BBC3* | down |  |
| *CDC6, CDK1* | up | Negative regulation of Cell cycle |
| *MMP3, CCL20, CXCL1* | down | TNF signaling pathway |
| *HIF1A* | up | HIF-1 signaling pathway |
| *MMP1, MMP3, MMP13, CCL20* | down | IL-17 signaling pathway |
| *COMP, COL2A1* | up | ECM-receptor interaction |
| *TGFB2* | up | MAPK signaling pathway |
| *FGFR1* | down |  |
| *CCL5, CCL20, IL8, IL6R* | down | Vital Protein Interaction |
| *COMP, COL2A1, IGF1* | up | Focal adhesion |
| *TGFB2, TGFB1, IGF1* | up | FoxO signaling pathway |
| *TGFB2, TGFB1, FOXM1* | up | Cellular senescence |
| *COMP, COL2A1, IGF1* | up | PI3K-Akt signaling pathway |
| *FGF* | down |  |
